# Real-World Outcomes of Direct-Acting Antiviral Treatment and Retreatment in United Kingdom–Based Patients Infected With Hepatitis C Virus Genotypes/Subtypes Endemic in Africa^[Author-notes jiab110-FM1]^

**DOI:** 10.1093/infdis/jiab110

**Published:** 2021-03-01

**Authors:** Elihu Aranday-Cortes, C Patrick McClure, Christopher Davis, William L Irving, Kazeem Adeboyejo, Lily Tong, Ana da Silva Filipe, Vattipally Sreenu, Kosh Agarwal, David Mutimer, Benjamin Stone, Matthew E Cramp, Emma C Thomson, Jonathan K Ball, John McLauchlan

**Affiliations:** MRC–University of Glasgow Centre for Virus Research, Glasgow, United Kingdom; National Institute for Health Research Nottingham Biomedical Research Centre, Nottingham University Hospitals National Health Service Trust and University of Nottingham, Nottingham, United Kingdom; Wolfson Centre for Emerging Virus Research, University of Nottingham, Nottingham, United Kingdom; School of Life Sciences, Faculty of Medicine and Health Sciences, University of Nottingham, Nottingham, United Kingdom; MRC–University of Glasgow Centre for Virus Research, Glasgow, United Kingdom; National Institute for Health Research Nottingham Biomedical Research Centre, Nottingham University Hospitals National Health Service Trust and University of Nottingham, Nottingham, United Kingdom; Wolfson Centre for Emerging Virus Research, University of Nottingham, Nottingham, United Kingdom; School of Life Sciences, Faculty of Medicine and Health Sciences, University of Nottingham, Nottingham, United Kingdom; National Institute for Health Research Nottingham Biomedical Research Centre, Nottingham University Hospitals National Health Service Trust and University of Nottingham, Nottingham, United Kingdom; Wolfson Centre for Emerging Virus Research, University of Nottingham, Nottingham, United Kingdom; School of Life Sciences, Faculty of Medicine and Health Sciences, University of Nottingham, Nottingham, United Kingdom; Olabisi Onabanjo University, Ago Iwoje, Nigeria; MRC–University of Glasgow Centre for Virus Research, Glasgow, United Kingdom; MRC–University of Glasgow Centre for Virus Research, Glasgow, United Kingdom; MRC–University of Glasgow Centre for Virus Research, Glasgow, United Kingdom; Institute of Liver Studies, Kings College Hospital National Health Service Foundation Trust, London, United Kingdom; Queen Elizabeth Hospital and University of Birmingham, Birmingham, United Kingdom; Department of Infection and Tropical Medicine, Sheffield Teaching Hospitals National Health Service Foundation Trust, Sheffield, United Kingdom; South West Liver Unit, Derriford Hospital and Peninsula Schools of Medicine and Dentistry, Plymouth, United Kingdom; MRC–University of Glasgow Centre for Virus Research, Glasgow, United Kingdom; National Institute for Health Research Nottingham Biomedical Research Centre, Nottingham University Hospitals National Health Service Trust and University of Nottingham, Nottingham, United Kingdom; Wolfson Centre for Emerging Virus Research, University of Nottingham, Nottingham, United Kingdom; School of Life Sciences, Faculty of Medicine and Health Sciences, University of Nottingham, Nottingham, United Kingdom; MRC–University of Glasgow Centre for Virus Research, Glasgow, United Kingdom

**Keywords:** HCV, direct-acting antiviral, DAA, treatment outcomes, Africa, viral genotypes/subtypes

## Abstract

**Background:**

Chronic hepatitis C virus (HCV) infection affects 71 million individuals, mostly residing in low- and middle-income countries (LMICs). Direct-acting antivirals (DAAs) give high rates of sustained virological response (SVR) in high-income countries where a restricted range of HCV genotypes/subtypes circulate.

**Methods:**

We studied United Kingdom–resident patients born in Africa to examine DAA effectiveness in LMICs where there is far greater breadth of HCV genotypes/subtypes. Viral genome sequences were determined from 233 patients.

**Results:**

Full-length viral genomic sequences for 26 known subtypes and 5 previously unidentified isolates covering 5 HCV genotypes were determined. From 149 patients who received DAA treatment/retreatment, the overall SVR was 93%. Treatment failure was associated primarily with 2 subtypes, gt1l and gt4r, using sofosbuvir/ledipasvir. These subtypes contain natural resistance-associated variants that likely contribute to poor efficacy with this drug combination. Treatment failure was also significantly associated with hepatocellular carcinoma.

**Conclusions:**

DAA combinations give high SVR rates despite the high HCV diversity across the African continent except for subtypes gt1l and gt4r, which respond poorly to sofosbuvir/ledipasvir. These subtypes are widely distributed across Western, Central, and Eastern Africa. Thus, in circumstances where accurate genotyping is absent, ledipasvir and its generic compounds should not be considered as a recommended treatment option.

## Teaser

We examined hepatitis C virus (HCV)–infected patients born in Africa but resident in the United Kingdom for treatment outcomes for direct-acting antiviral therapy. The unusual HCV subtypes identified in many patients successfully responded to treatment except for 2 subtypes.

Direct-acting antivirals (DAAs) capable of clearing chronic hepatitis C virus (HCV) infection from 90%–95% of treated individuals are a cornerstone of the World Health Organization (WHO) strategy to eliminate viral hepatitis as a public health concern by 2030 [1]. Consequently, many high-income countries (HICs) have implemented action plans for controlling infection and transmission to achieve HCV eradication. By contrast, low- and middle-income countries (LMICs) face obstacles and challenges limiting their ability to implement similar strategies [2]. Moreover, clinical trials and data on DAA efficacy are almost exclusively derived from studies in HICs where circulating HCV genotypes (gt)/subtypes are more restricted than those in LMICs [3–6]. In particular, most subtypes of HCV gt4, gt5, and gt6 have been largely neglected, yet they represent about 15% of chronic HCV infections [7].

We recently reported a substantial gap in our knowledge of HCV genomic sequences circulating in LMICs across large geographic areas [8]. In addition, we and others have identified HCV gt1 and gt4 subtypes in infected patients of African origin that do not respond to DAA therapy as well as subtypes typically transmitted in HICs; treatment failure using a sofosbuvir (SOF)/ledipasvir (LDV) combination has been particularly evident with HCV subtypes gt1l [9, 10] and gt4r [10–14]. Reduced DAA efficacy for these subtypes is frequently related to polymorphisms in DAA target proteins (herein defined as resistance-associated variants [RAVs]), which can give inherent resistance to certain DAA combinations.

In this study, we address the issue of treatment outcomes in response to DAA therapy in HCV-infected individuals originating from Africa but residing in the United Kingdom (UK). We also aimed to increase the number of available complete HCV genomic sequences derived from African patients, as there is a lack of such genetic data in public databases.

## METHODS

### Study Design and Patients

HCV-infected patients were enrolled into the HCV Research UK cohort at clinical sites in the UK [15]. All patients whose country of birth was in Africa were included in this study. Further details of the HCV Research UK clinical database are given in the Supplementary Methods. The study conforms to the ethical guidelines of the 1975 Declaration of Helsinki as reflected in a priori approval by the institution’s human research committee. Ethics approval for HCV Research UK was given by National Research Ethics Service (NRES) Committee East Midlands–Derby 1 (Research Ethics Committee reference 11/EM/0314).

### Next-Generation Sequencing by Metagenomics and Target Enrichment

The next-generation sequencing (NGS) methods for determining HCV viral sequences by metagenomics and target enrichment have been published previously [16] and are described in the Supplementary Methods.

### HCV Sequence and Variant Analysis

Next-generation sequencing data quality was assessed using FastQC (http://www.bioinformatics.babraham.ac.uk/projects/fastqc/), and low-quality bases (Phred score <30 or read length <50 nucleotides) were trimmed using Trim Galore! script (https://www.bioinformatics.babraham.ac.uk/projects/trim_galore/). Cleaned reads were submitted for de novo assembly using SPAdes [17]. Where de novo assembly failed to generate a full-length contig, reads were mapped to the closest reference genome. Reference genomes were selected by a kmer-based approach using unique kmers from all HCV genotypes. Reads were mapped to the reference genome (or to de novo contigs) using Tanoti read mapper (https://bioinformatics.cvr.ac.uk/software/tanoti/). Any minor variants associated with DAA resistance were identified using the GLUE software package *samReporter* with default settings [18, 19].

### Statistical Analysis

Statistical analyses were performed using Stata version 10 software. Continuous variables were reported as median (and interquartile range) and analyzed by the Mann–Whitney *U* test for univariate analysis. Categorical univariate analysis was performed using Fisher exact test. Multivariate analysis was carried out using logistic regression. Variables with a *P* value of <.1 in univariate analysis were included in multivariate analysis.

## RESULTS

### Demographics of Subjects Originating From Africa in the Cohort

Within the HCV Research UK cohort [15], 319 patients were born in 32 countries across Africa. Analysis of this African group revealed that 66.5% were male (Table 1) with an age range of 24–88 years, averaging 59 years. The most frequently cited probable sources of infection were “born abroad” (40%) and “blood/blood products” (17%) (Table 1). “Born abroad” was included as it was significantly associated as a risk factor for infection in a previous study [20]. Only 10% of individuals probably acquired infection through injection drug use, considerably lower than that for white UK nationals from the HCV Research UK cohort (n = 5360/8419 [64%]). Moreover, 44 cases were cited as “other” (14% of the cohort) as a probable source of infection; within this category, there was a range of possible sources of infection, but none were reported as having acquired infection in the UK (Supplementary Table 1). Indeed, for 11 individuals, country of birth was recorded specifically as the likely location for acquiring infection (Table 1 and Supplementary Table 1).

**Table 1.** Demographics of Hepatitis C Virus–Infected Individuals Originating From Africa (N = 319)

Characteristic	No.	(%)
Sex		
Male	212	(66.5)
Female	105	(33)
Not known	2	(<1)
Cirrhosis (n = 131)^a^		
No decompensation	53	(40.5)
Decompensated	42	(32)
HCC	25	(19)
Decompensated + HCC	11	(8.5)
Probable source of infection (N = 319)		
Born abroad	127	(40)
Blood/blood product	54^b^	(17)
Injection drug use	33	(10)
Known HCV-positive partner	6	(2)
Perinatal exposure	1	(<0.1)
Other	44	(14)
No known risk factor	29	(9)
Data incomplete	7	(2)
No data entry	18	(5.5)

Abbreviations: HCC, hepatocellular carcinoma; HCV, hepatitis C virus.

^a^Stratification of HCV-infected cases with cirrhosis by disease type.

^b^For 2 individuals, country of birth was recorded as the likely location for acquiring infection.

A high proportion of the African cohort had cirrhosis (n = 131 [41%]) and, within this group, decompensated disease was found in 42 individuals (32%). In addition, 25 cases (19%) were diagnosed with hepatocellular carcinoma (HCC) (Table 1). The percentage of UK-born individuals of white ethnicity in the HCV Research UK database with cirrhosis was 37% (n = 3141/8419). Within this group, the percentage with more severe liver disease was considerably less (n = 1203/3141 [38%]; Supplementary Table 2) compared to the African group (n = 78/131 [59.5%]; Table 1).

### Identification of HCV Genotypes and Subtypes by Unbiased NGS and Target Enrichment

Using a NGS target enrichment approach (described in the Supplementary Methods) [16], we generated sequence data covering the entire HCV coding region for samples from 233 individuals (Supplementary Table 3); viral sequence data were determined from another 14 samples collected from patients after they underwent unsuccessful DAA therapy. Samples that did not yield viral sequence data were mostly obtained either during or after antiviral therapy (64/86 [75%]), at which point either very low or undetectable viral loads would be evident in circulating blood.

**Figure 1.**
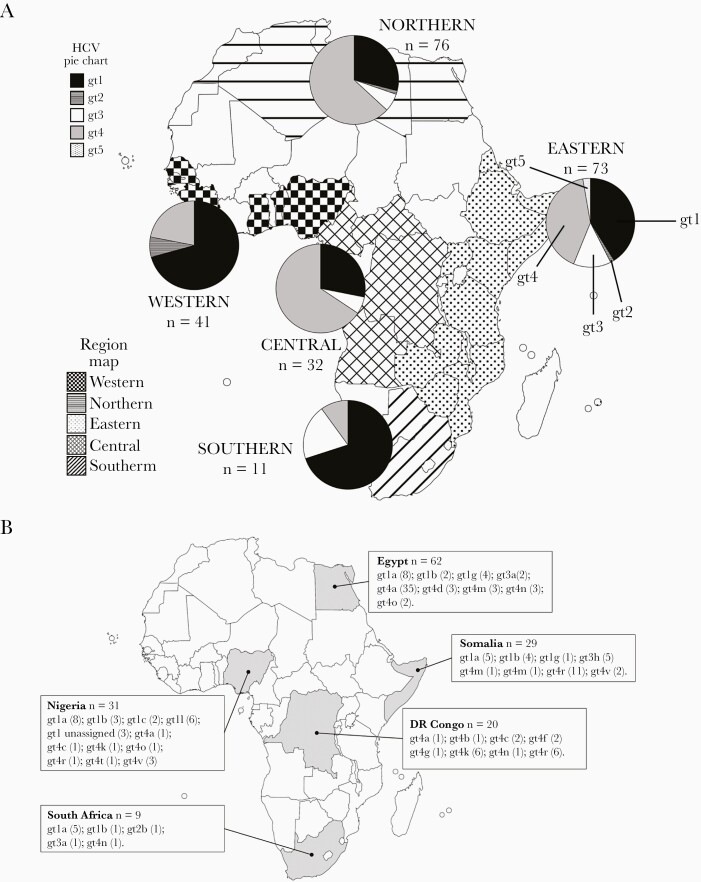
*A*, Distribution of hepatitis C virus (HCV) genotypes from individuals originating from Northern, Western, Central, Eastern, and Southern Africa. *B*, Numbers of HCV genotypes and subtypes identified in individuals originating from the most highly represented countries in Northern, Western, Central, Eastern, and Southern Africa.

Using maximum likelihood phylogenetic analysis, we detected 7 gt1 subtypes (and 4 gt1 unassigned isolates), 3 gt2 subtypes (and 1 gt2 unassigned isolate), 2 gt3 subtypes, 13 gt4 subtypes, and 2 gt5a sequences; unassigned isolates differed from classified HCV subtypes by at least 15% at the nucleotide level [21]. Compared to publicly available data, our analysis considerably increases the genomic information for 8 subtypes (gt1g, gt1l, gt3h, gt4a, gt4c, gt4k, gt4n, and gt4r). The distribution and occurrence of the HCV genotypes and subtypes identified in each individual and their country of origin are shown in Figure 1 and Supplementary Tables 3 and 4. From analysis of the geographical locations of all confirmed subtypes for HCV gt1–gt4 available in the published literature [21–25], there were only 7 subtypes previously found in Africa that were absent in our sequencing dataset (Supplementary Table 4).

The most highly represented countries in each geographic African region for sequence data were Egypt (n = 62; Northern Africa), Nigeria (n = 31; Western Africa), Democratic Republic of the Congo (DRC)/Republic of Congo (n = 20; Central Africa), Somalia (n = 29; Eastern Africa), and South Africa (n = 9; Southern Africa) (Figure 1A and 1B). HCV subtypes gt1a and gt1b were ubiquitous in patients born across Africa, with either subtype found in individuals from 18 of 27 countries (67%). Genotype 4a was the most common subtype in Egyptian individuals, as previously reported [26, 27]. Aside from gt1a, gt1b, and gt4a, the most frequently detected subtypes were gt3a and gt4r, which were found in individuals from 9 of 27 and 7 of 27 countries, respectively. Genotype 4r was present in patients originating from Western Africa (Nigeria), Central Africa (Central African Republic and DRC/Republic of Congo), and Eastern Africa (Burundi, Eritrea, Ethiopia, and Somalia) (Figure 1A and 1B). Strikingly, 38% (11/29) of sequences from Somalian patients were gt4r (Figure 1B). Therefore, gt4r is likely to be distributed across large areas of Africa and, assuming that most individuals were infected in their originating country, may dominate in certain countries. Genotype 3h was also detected in patients from Somalia and Zimbabwe and hence may be the most common gt3 subtype circulating in certain African countries. Indeed, gt3h was the second most prevalent subtype in Somalian individuals after gt4r. In addition, our sequence studies identified 5 unassigned strains for gt1 and gt2, which were detected in patients from Nigeria (n = 3), Cameroon (n = 1), and Ghana (n = 1) (Supplementary Table 3).

We compared the genotypes/subtypes identified by NGS analysis with those recorded by the clinical site that used commercial kits (such as the INNO-LiPA test [Innogenetics]). We obtained genotype and subtype data for both commercial assays and NGS for 133 and 60 samples, respectively (genotype and subtype). HCV genotype was identical in 124 samples (93%), but the percentage of concordant HCV subtypes was much lower (n = 41/60 samples [68%]). The majority of mismatches corresponded to subtypes that are not typically identified or differentiated in commercial kits (data not shown).

### Outcomes of DAA Therapy

We evaluated treatment outcomes for all DAA drug combinations used to treat the cohort, yielding data on 149 patients. This group included 12 patients who had received prior DAA therapy on 1 (n = 11) or 2 (n = 1) occasions. Hence, there was a total of 162 treatment episodes recorded (Table 2). Two patients were lost to follow-up or died before treatment outcome was known but were included in the overall analysis.

**Table 2.** Direct-Acting Antiviral–Only Regimens and Outcomes for Individuals Originating From Africa

DAA Regimen	Total	Treatment Regimen Outcomes (n = 162)				
		SVR, No. (%)	Responder-Relapser	Nonresponder	Lost to Follow-up	Died Before Outcome Known
All	162	136 (84)	21	3	1	1
OBV/PTV/RTV	15	14 (93)	1	0	0	0
OBV/PTV/RTV/DSV	24	24 (100)	0	0	0	0
GLE/PIB	7	6 (86)	0	1	0	0
SOF	4	3 (75)	1	0	0	0
SOF/LDV	71	55 (77)	14	1	0	1
SOF/DCV	14	12 (86)	2	0	0	0
SOF/DCV or LDV	3	3 (100)	0	0	0	0
SOF/VEL	8	6 (75)	1	1	0	0
SOF/VEL/VOX	4	3 (75)	1	0	0	0
GRZ/ELB	12	10 (83)	1	0	1	0

Abbreviations: DAA, direct-acting antiviral; DCV, daclatasvir; DSV, dasabuvir; GLE, glecaprevir; GRZ, grazoprevir; LDV, ledipasvir; OBV, ombitasvir; PIB, pibrentasvir; PTV, paritaprevir; RTV, ritonavir; SOF, sofusbuvir; SVR, sustained virological response; VEL, velpatasvir; VOX, voxilaprevir.

From univariate analysis, presence of HCC (*P* = .006) was significantly associated with DAA failure; a higher failure rate was noted also for those with decompensated liver disease (*P* = .061; Supplementary Table 5). No other demographic or clinical characteristics, including HIV infection, were associated with failed response to DAA therapy. From combining all types of therapy (both interferon [IFN] and DAA based), treatment-naive patients were significantly more likely to respond compared with treatment-experienced individuals (Supplementary Table 5). Stratifying the various treatment regimens according to either IFN or DAA targeted at HCV proteins (NS3, NS5A, and NS5B) revealed that prior exposure to antivirals against NS5A and NS5B was significantly associated with subsequent treatment failure (Supplementary Table 5).

To identify the basis of this finding, we examined the viral sequence data for the group that received DAA therapy. In total, viral sequences were available for 131 patients who had received 144 treatment episodes. The most striking observation from analyzing the different DAA combinations for these patients was low SVR rates for gt1l and gt4r with NS5A/NS5B antiviral combinations (0% and 44%, respectively; Table 3). Statistical analysis of both HCV subtypes combined showed that a low SVR was highly significant in both univariate and multivariate analysis compared to other subtypes (Supplementary Table 5). By comparison, high SVR rates were achieved for other subtypes that are common across Africa but found far less frequently in HCV-infected populations in other continents.

**Table 3.** Sustained Virologic Response Rates by Hepatitis C Virus Genotype and Direct-Acting Antiviral Regimen for Patients With Next-Generation Sequencing Data

HCV Genotype	SVR, no./No. (%)			
	All DAA Regimens	NS3/NS5A DAA Regimens	NS5A/NS5B DAA Regimens	NS3/NS5A/NS5B DAA Regimens
All	119/144 (83)	27/31 (87)	67/87 (77)	25/26 (96)
gt1a	23/26 (88)	3/5 (60)	16/17 (94)	4/4 (100)
gt1b	16/17 (94)	3/3 (100)	8/9 (89)	5/5 (100)
gt1l	3/10 (30)	1/1 (100)	0/7 (0)	2/2 (100)
gt3a	7/9 (90)	1/1 (100)	6/8 (75)	0
gt4a	25/27 (93)	10/11 (91)	12/13 (92)	3/3 (100)
gt4d	4/4 (100)	0	3/3 (100)	1/1 (100)
gt4r	9/15 (60)	2/2 (100)	4/9 (44)	3/4 (75)

Abbreviations: DAA, direct-acting antiviral; gt, genotype; HCV, hepatitis C virus; SVR, sustained virological response.

### Virological Characteristics of Responder-Relapsers/Nonresponders to DAA-Based Therapy and Retreatment Outcomes

In terms of virological outcomes (ie, excluding patients either lost to follow-up or who died before outcome was known), there were 24 treatment episodes with DAA that led to treatment failure (Table 2) in 19 patients. Final records for these patients showed that 9 were successfully retreated. Four patients who were retreated did not achieve SVR and, therefore, 10 patients remained viremic (Table 4). Five patients were retreated with the same drug combination and only 2 (both infected with gt3a) achieved SVR. The other 3 patients were infected with either gt1l (n = 2) or gt4r (n = 1). Six of 7 patients who received retreatment with more potent DAA combinations, either glecaprevir (GLE)/ pibrentasvir (PIB) or SOF/velpatasvir (VEL)/voxilaprevir (VOX), achieved SVR; the 1 retreatment failure in this category was infected with gt4r and received SOF/VEL/VOX (Table 4).

**Table 4.** Characteristics of Patients With Unsuccessful Direct-Acting Antiviral Treatment Episodes and Outcomes of Retreatment

Patient	HCV Genotype	Liver Disease			DAA Treatment Episodes and Outcomes			
		Cirrhosis	Decompensated	HCC	Rx1	Outcome	Rx2	Outcome
Patients who did not achieve SVR								
1	gt1a	Yes	No	No	GLE/PIB	NR	No	…
2	gt1-	No	No	No	GRZ/ELB/RBV	RR	No	…
3	gt1l	Yes	No	No	SOF/LDV/RBV	RR	No	…
4	gt1l	Yes	Yes	Yes	SOF/LDV/RBV	RR	SOF/LDV/RBV	RR
5	gt1l	Yes	Yes	Yes	SOF/DCV	RR	SOF/DCV	RR
6	gt3h	Yes	No	No	SOF/VEL/RBV	RR	No	…
7	gt4a	Yes	No	No	SOF/LDV	RR	No	…
8	gt4r	Yes	Yes	Yes	SOF/LDV/RBV	RR	No	…
9	gt4r	Yes	Yes	No	SOF/LDV/RBV	NR	SOF/LDV/RBV	RR
10	gt4r	No	No	No	SOF/LDV	RR	SOF/VEL/VOX	RR
Patients who did achieve SVR on retreatment								
11	gt1a	No	No	No	SOF/LDV	RR	SOF/VEL/VOX	SVR
12	gt1b	No	No	No	SOF/RBV	RR	SOF/VEL/VOX	SVR
13	gt1c	No	No	No	SOF/LDV/RBV	RR	Clinical trial^a^	SVR
14	gt1l	Yes	Yes	Yes	SOF/LDV/RBV^b^	RR	GLE/PIB	SVR
15	gt3a	Yes	Yes	No	SOF/LDV/RBV	RR	SOF/LDV/RBV	SVR
16	gt3a	Yes	Yes	Yes	SOF/LDV/RBV	RR	SOF/LDV/RBV	SVR
17	gt4a	Yes	No	No	OBV/PTV/RTV	RR	GLE/PIB	SVR
18	gt4o	Yes	No	No	SOF/VEL	NR	GLE/PIB	SVR
19	gt4r	No	No	No	SOF/LDV	RR	SOF/VEL/VOX	SVR

Abbreviations: DAA, direct-acting antiviral; DCV, daclatasvir; ELB, elbasvir; GLE, glecaprevir; GRZ, grazoprevir; gt1-, gt1 unassigned subtype; HCC, hepatocellular carcinoma; HCV, hepatitis C virus; gt, genotype; LDV, ledipasvir; NR, nonresponder; OBV, ombitasvir; PIB, pibrentasvir; PTV, paritaprevir; RBV, ribavirin; RR, responder-relapser; RTV, ritonavir; Rx, prescribed treatment; SOF, sofusbuvir; SVR, sustained virological response; VEL, velpatasvir; VOX, voxilaprevir.

^a^DAA treatment used in clinical trial was not known.

^b^Patient received a combination of SOF/LDV/RBV twice and was a responder-relapser on both occasions.

### Sequences in NS5A for Subtypes gt1l and gt4r Potentially Associated With Lower Response to DAA Therapy

Given the frequency of gt1l and gt4r treatment failure, we examined all individuals in the cohort infected with these HCV subtypes for both their viral sequences and outcomes from antiviral therapy. Seven patients were infected with gt1l (Supplementary Table 4), 6 of whom received DAA therapy (Table 5). Twenty-six patients were infected with gt4r (Supplementary Table 4), 12 of whom received at least 1 episode of DAA therapy (Table 5). Combining the data for gt1l and gt4r indicated that there were 13 instances of DAA treatment failure, 10 of which resulted from SOF/LDV treatment; the remaining treatment failures arose from use of SOF/daclatasvir (DCV) (n = 2) for a gt1l-infected individual and SOF/VEL/VOX for retreatment in a gt4r infection (Table 4). Previously, we have shown that sequences from these subtypes can share a common motif in NS5A, M^28^R^30^M^31^, that could give resistance to NS5A inhibitors and thereby reduce effectiveness of DAA therapy [10]. From examining the NS5A coding region for all gt1l and gt4r viral sequences in the cohort, we found that gt1l had 2 patterns at positions 28, 30, and 31; methionine was invariant at positions 28 and 31, while position 30 encoded either arginine or glutamine (Table 5). The same positions in gt4r had more complex amino acid combinations with 6 distinct patterns of residues identified in the 11 patients who received DAA treatment (Table 5). Position 30 encoded an arginine residue that was invariant, while position 31 encoded predominantly leucine with methionine present at a lower frequency. The highest variability was observed at position 28 with methionine found at highest frequency, but 4 other amino acids were also encoded at this position (Table 5).

**Table 5.** NS5A Sequences at Sites Associated With Direct-Acting Antiviral (DAA) Resistance and Outcomes of DAA Treatment Regimens for Individuals Infected With Genotype 1l or Genotype 4r

NS5A Position			Frequency	NS3/NS5A Rx		NS5A/NS5B Rx		NS3/NS5A/NS5B Rx	
28	30	31		N_Rx_	N_SVR_	N_Rx_	N_SVR_	N_Rx_	N_SVR_
*gt1l*			n = 6	1	1	7	0	2	2
M	Q	M	n = 3	0	0	3	0	1	1
M	R	M	n = 3	1	1	4	0	1	1
*gt4r*			n = 12	2	2	9	4	4	3
M	R	L	n = 6	1	1	4	2	2	2
M	R	M	n = 1	0	0	2	0	0	0
V	R	L	n = 2	0	0	2	1	1	0
F	R	L	n = 1	0	0	1	1	0	0
I	R	L	n = 1	0	0	0	0	1	1
T	R	L	n = 1	1	1	0	0	0	0

Underlined amino acids indicate possible resistance-associated variants.

Abbreviations: N_Rx_, number receiving prescribed treatment; N_SVR_, number achieving sustained virological response; Rx, prescribed treatment.

## DISCUSSION

Our study had 2 major objectives; first, to analyze DAA treatment outcomes for individuals originating from countries across Africa, and second, to determine the HCV sequences circulating in the cohort. Given the sparsity of viral sequences at RAV positions and real-world outcomes of DAA therapy from African individuals, our study addresses a critical gap in such information from LMICs [8].

The cohort totaled 319 individuals, originating from 32 African countries. This gives a broad geographical spread with larger groups coming from Egypt (Northern Africa), Nigeria (Western Africa), DRC/Republic of Congo (Central Africa), Somalia (Eastern Africa), and South Africa (Southern Africa). For most individuals, HCV infection was likely acquired in their country of origin given their more advanced stage of liver disease (Table 1 and Supplementary Table 2) and the diversity of genotypes and subtypes represented in our analysis (Supplementary Tables 3, 4, and 6), as reported previously for African individuals [22–25, 28]. In addition, some individuals reported likely transmission in their country of origin (Supplementary Table 1). We cannot exclude the possibility that a proportion of infections occurred in the UK or in countries from which individuals did not originate. In the UK, HCV is predominantly found in people who inject drugs (PWID), and the genotypes observed are almost exclusively gt1 (53%) and gt3 (41%; Supplementary Table 6). By contrast, African individuals had much lower occurrence of gt3 infection (8%) but far higher prevalence of gt4 infection (47% compared to 0.5% in the UK PWID population; Supplementary Table 6). Moreover, the subtypes identified in the UK PWID population are typically gt1a, gt1b, and gt3a (data not shown), but there is greater diversity of gt1 subtypes in the African group (Supplementary Tables 3 and 4) and there were no cases of gt3h in the UK PWID cohort. We did attempt to distinguish gt1a, gt1b, and gt3a strains in African individuals compared to UK-based infections, but phylogenetic analysis did not discriminate between possible UK and African strains (data not shown). Thus, it is highly probable that for the African group, HCV infection occurred in their country of origin. Far larger studies would be required to determine whether the higher prevalence of liver disease was associated with any virological factors given the infrequency of many of the subtypes outside Africa.

Almost half of the cohort was treated with 9 different DAA regimens including NS3 protease + NS5A inhibitors, NS5A + NS5B inhibitors, and drug combinations against all 3 viral targets. The most frequently prescribed DAA therapies were SOF-based regimens (104/162 [64%]). SOF was occasionally used as mono-DAA therapy with ribavirin (RBV) but was mostly prescribed in dual combination with NS5A inhibitors with or without RBV (LDV, VEL, or DCV) or triple combination (VEL/VOX) (Table 2). The overall SVR for SOF-based treatment was 79% (82/104). This relatively low SVR rate was primarily a consequence of relapse with SOF/LDV in gt1l- and gt4r-infected patients (Table 2). These findings confirm previous reports from ourselves and others on the lower SVR rates achieved with SOF/LDV therapy for both of these subtypes [9–14]. By contrast, 8 of 9 patients (89%) infected with either gt1l or gt4r achieved SVR following treatment with either NS3/NS5A combinations or triple SOF/VEL/VOX treatment.

Aside from gt1l and gt4r, the SVR for other gt1 and gt4 subtypes was 97%. This includes gt4d, another unusual subtype associated with treatment failure with ombitasvir (OBV)/paritaprevir (PTV)/ritonavir (RTV) in a clinical trial [29]. There were 4 gt4d-infected patients in our study, all of whom achieved SVR with OBV/PTV/RTV (n = 1) and SOF-based therapy (n = 3). Thus, it is likely that there are few gt1 and gt4 subtypes, other than gt1l and gt4r, circulating in Africa that would not give high SVR rates for the various DAA therapies. Nonetheless, exceptions could arise such as the unassigned gt1 strain from a Nigerian patient who had a T28 + S30 + N93 amino acid combination in NS5A and was a responder-relapser to grazoprevir/elbasvir treatment (patient 2 in Table 4). Moreover, the gt1a-infected case, who was classified as a nonresponder to GLE/PIB, had a natural M31 RAV in NS5A that appears infrequently in gt1a sequences but could arise more frequently in certain LMIC populations that have not been extensively analyzed. Thus, continued surveys of viral sequences in Africa and outcomes from therapy would be beneficial.

Thirteen patients in our cohort who were not successfully cured by initial DAA therapy received retreatment, with 9 achieving SVR. Aside from 1 individual who relapsed on retreatment, the more potent DAA combinations containing GLE/PIB and SOF/VEL/VOX achieved cure in 6 patients. SOF/VEL/VOX retreatment was unsuccessful in 1 individual with gt4r (Table 4) who had received previous unsuccessful SOF/LDV therapy. The reason for retreatment failure with SOF/VEL/VOX is not known since there were no serial samples available following SOF/LDV treatment. In addition, the patient did not have underlying severe liver disease. We also did not find any potential resistance-associated substitutions (RASs) in the NS3 protein that could explain lack of efficacy for VOX. It is possible that initial SOF treatment could have led to emergence of an S282T RAS in NS5B, which would yield high levels of resistance. From previous reports, S282T appears to emerge at higher frequency with gt4r than other HCV genotypes and subtypes [10, 13]. This RAS did emerge transiently in 1 patient in our study (patient 9 in Table 4) following initial SOF/LDV treatment. We also found a V321I variant [12] in the NS5B sequences of 6 gt4r-infected patients who received SOF treatment; 3 patients achieved SVR whereas the remainder relapsed from therapy. Notably, in the French study that documented successful retreatment outcomes in 7 patients infected with gt4r, again GLE/PIB and SOF/VEL/VOX were used in most retreatment regimens [13]. Overall, studies from our group and others agree that SOF/LDV therapy is suboptimal and should not be recommended in regions with potentially significant prevalence of gt1l and gt4r infection. This recommendation is reinforced by a recent report using subgenomic replicons containing gt1l and gt4r NS5A sequences, which shows that variants at positions 30 and 31 identified here increase resistance to LDV [30]. It is worth noting that African [14], UK [9, 10], and French studies [11–13] have now highlighted difficulties with treating patients of African origin infected with these subtypes using SOF/LDV therapy. Therefore, treating this patient group with a SOF/LDV combination in high-income settings should be avoided unless robust HCV genotype/subtype assays are available. In resource-limited settings where there is considerable diversity of HCV genotypes and subtypes that could include gt1l and gt4r, more potent pangenotypic DAA combinations should prove effective as either initial or rescue therapy, thereby obviating the need for sophisticated assays for viral genotyping and subtyping. During the review of this manuscript, the most recent European Association for the Study of the Liver (EASL) guidelines excluded SOF/LDV as recommended therapy, but do highlight the need for additional treatment outcome data for unusual HCV subtypes such as gt1l and gt4r with more potent DAA combinations [31].

In conclusion, our study describes HCV subtypes and viral sequences circulating in countries for which there are very limited data, and complements our recent report describing gt4 subtypes in Uganda and DRC as well as novel gt7 strains [22]. Crucially, we show that gt4r is not a “rare” subtype across large geographical regions in Africa. Indeed, excluding Egypt where gt4a dominates [26, 32], gt4r is the most prevalent gt4 subtype in our study and therefore may be highly prevalent in certain African countries. Moreover, the extent of gt1l infection in Nigeria and other unassigned gt1 strains has not been fully evaluated. As highlighted in EASL guidelines, it would be prudent to continue to record outcome data to determine the optimum regimens for use in regions where such subtypes circulate.

## Supplementary Data

Supplementary materials are available at *The Journal of Infectious Diseases* online. Consisting of data provided by the authors to benefit the reader, the posted materials are not copyedited and are the sole responsibility of the authors, so questions or comments should be addressed to the corresponding author.

jiab110_suppl_Supplementary_Table_1

jiab110_suppl_Supplementary_Table_2

jiab110_suppl_Supplementary_Table_3

jiab110_suppl_Supplementary_Table_4

jiab110_suppl_Supplementary_Table_5

jiab110_suppl_Supplementary_Table_6

jiab110_suppl_Supplementary_Methods

## Notes

***Acknowledgments.*** We gratefully acknowledge the provision of clinical data and samples from the HCV Research UK cohort and thank the patients who consented to be enrolled in the cohort. We also thank Dr Josh Singer (University of Glasgow) for assistance with analysis using the HCV-GLUE online resource (http://hcv.glue.cvr.ac.uk/#/home).

***Financial support.*** This work was funded by the UK Medical Research Council (MC_UU_12014/1). HCV Research UK received support from the Medical Research Foundation (C0365). E. C. T. was also funded by the Wellcome Trust (102789/Z/13/A). K. Ad. was supported by the Tertiary Education Trust Fund and the National Institute for Health Research Nottingham Biomedical Research Centre.
