# Supplementary material for: Real-World Outcomes of Direct-Acting Antiviral Treatment and Retreatment in United Kingdom–Based Patients Infected With Hepatitis C Virus Genotypes/Subtypes Endemic in Africa
Source: J Infect Dis. 2021 Mar 1;226(6):995–1004. doi: 10.1093/infdis/jiab110 (PMC9492310; doi:10.1093/infdis/jiab110)
Supplement: jiab110_suppl_Supplementary_Table_1 [file jiab110_suppl_supplementary_table_1.docx]

**Supplementary Table 1.** Possible routes of HCV infection for individuals recorded as ‘Other’ (n=44) as a route of infection in the HCV Research UK database.

| **Possible route of infection** | **n** | **Transmission occurred in country of origin (n)** |
| --- | --- | --- |
| Surgical or dental procedures | 11 | 6 |
| Vaccination or needlestick injury | 8 | 2 |
| Healthcare worker or occupational risk | 3 | 1 |
| Possible HCV-infected sexual partner | 3 | 0 |
| Tattooing | 1 | 0 |
| Not known | 18 | 0 |
