# Supplementary material for: Real-World Outcomes of Direct-Acting Antiviral Treatment and Retreatment in United Kingdom–Based Patients Infected With Hepatitis C Virus Genotypes/Subtypes Endemic in Africa
Source: J Infect Dis. 2021 Mar 1;226(6):995–1004. doi: 10.1093/infdis/jiab110 (PMC9492310; doi:10.1093/infdis/jiab110)
Supplement: jiab110_suppl_Supplementary_Table_2 [file jiab110_suppl_supplementary_table_2.docx]

|  | **Cirrhosis (n=3141)^a^** | | | |  |
| --- | --- | --- | --- | --- | --- |
|  | *Cirrhosis, no decompensation* | *Decompensated* | *HCC* | *Decompensated + HCC* | |
| n  (%) | 1938  (62%) | 680  (21.5%) | 301  (9.5%) | 222  (7%) | |

**Supplementary Table 2.** Liver disease characteristics in HCV-infected individuals of white ethnicity originating from the UK (n=8419).

^a^ Stratification of HCV-infected cases with cirrhosis by disease type.
