# Supplementary material for: Real-World Outcomes of Direct-Acting Antiviral Treatment and Retreatment in United Kingdom–Based Patients Infected With Hepatitis C Virus Genotypes/Subtypes Endemic in Africa
Source: J Infect Dis. 2021 Mar 1;226(6):995–1004. doi: 10.1093/infdis/jiab110 (PMC9492310; doi:10.1093/infdis/jiab110)
Supplement: jiab110_suppl_Supplementary_Table_3 [file jiab110_suppl_supplementary_table_3.docx]

**Supplementary Table 3.** Countries of origin of patients in the HCV Research UK cohort and HCV sequences identified in patients from each country as determined by NGS.

| **Region** | **Country of origin (n=32)** | **No of cases**  **(n=319)** | **HCV sequences (n=233)** | **Genotypes/subtypes^a^** |
| --- | --- | --- | --- | --- |
| Northern Africa | Algeria | 5 | 5 | gt1a (2); gt1b (2); gt3a (1) |
|  | Egypt | 82 | 62 | gt1a (8); gt1b (2); gt1g (4)^b^; gt3a (2); gt4a (35); gt4d (3); gt4m (3); gt4n (3); gt4o (2) |
|  | Libya | 4 | 1 | gt4o (1) |
|  | Morocco | 9 | 6 | gt1a (3); gt2c (1); gt3a (1); gt4a (1) |
|  | Tunisia | 3 | 2 | gt1d (1); gt3a (1) |
|  | ***Total*** | ***103*** | ***76*** |  |
| Western Africa | Benin | 1 | 0 |  |
|  | Gambia | 1 | 1 | gt1e (1) |
|  | Ghana | 9 | 8 | gt1a (3); gt1b (2); gt2c (1); gt2q (1); gt2 unassigned (1) |
|  | Guinea | 1 | 1 | gt1b |
|  | Nigeria | 38 | 31 | gt1a (8); gt1b (3); gt1c (2); gt1l (6); gt1 unassigned (3); gt4a (1); gt4c (1); gt4k (1); gt4o (1); gt4r (1); gt4t (1) gt4v (3) |
|  | Senegal | 1 | 0 |  |
|  | ***Total*** | ***51*** | ***41*** |  |
| Central Africa | Angola | 5 | 5 | gt1a (5) |
|  | Cameroon | 6 | 4 | gt1a (1); gt1e (1); gt1l (1); gt1 unassigned (1) |
|  | Central African Republic | 3 | 3 | gt3a (2); gt4r (1) |
|  | DRC/Republic of Congo | 24 | 20 | gt4a (1); gt4b (1); gt4c (2); gt4f (2); gt4g (1); gt4k (6); gt4n (1); gt4r (6) |
|  | Equatorial Guinea | 1 | 0 |  |
|  | ***Total*** | ***39*** | ***32*** |  |
| Eastern Africa | Burundi | 3 | 2 | gt4b (1); gt4r (1) |
|  | Eritrea | 9 | 7 | gt4d (2); gt4r (4); gt5a (1) |
|  | Ethiopia | 7 | 7 | gt1a (2); gt1b (1); gt4d (2); gt4r (2) |
|  | Kenya | 12 | 5 | gt1a (2); gt1b (1); gt3a (2) |
|  | Malawi | 1 | 0 |  |
|  | Mauritius | 8 | 2 | gt1a (1); gt1b (1) |
|  | Mozambique | 6 | 1 | gt4a (1) |
|  | Rwanda | 1 | 0 |  |
|  | Somalia | 39 | 29 | gt1a (5); gt1b (4); gt1g (1); gt3h (5); gt4m (1); gt4r (11); gt4v (2) |
|  | Tanzania | 4 | 4 | gt1a (2); gt3a (1); gt4d (1) |
|  | Uganda | 3 | 3 | gt1a (1); gt4v (2) |
|  | Zambia | 3 | 1 | gt1a (1) |
|  | Zimbabwe | 14 | 12 | gt1a (2); gt1b (5); gt1g (1); gt3h (2); gt4c (1); gt5a (1) |
|  | ***Total*** | ***110*** | ***73*** |  |
| Southern Africa | Botswana | 1 | 1 | gt1b |
|  | South Africa | 14 | 9 | gt1a (5); gt1b (1); gt2b (1); gt3a (1); gt4n (1) |
|  | Swaziland | 1 | 1 | gt3a (1) |
|  | ***Total*** | ***16*** | ***11*** |  |

^a^ Numbers in brackets indicate the number of sequences determined for each genotype/subtype.

**^b^** One gt1g-infected patient also had a minority of sequences mapping to gt4r; this could represent co-infection with both subtypes but for analytical purposes, the patient was considered as gt1g-infected.
