# Supplementary material for: Real-World Outcomes of Direct-Acting Antiviral Treatment and Retreatment in United Kingdom–Based Patients Infected With Hepatitis C Virus Genotypes/Subtypes Endemic in Africa
Source: J Infect Dis. 2021 Mar 1;226(6):995–1004. doi: 10.1093/infdis/jiab110 (PMC9492310; doi:10.1093/infdis/jiab110)
Supplement: jiab110_suppl_Supplementary_Table_4 [file jiab110_suppl_supplementary_table_4.docx]

**Supplementary Table 4.** HCV genotypes and subtypes identified in the cohort.

| **HCV genotype** | **HCV subtype** | **No of sequences** | **Absent African subtypes** |
| --- | --- | --- | --- |
| gt1 (n=97; 42%) | gt1a | 51(22%) | gt1h (Cameroon)^ref [25]^ |
|  | gt1b | 24 (10%) |  |
|  | gt1c | 2 (<1%) |  |
|  | gt1d | 1 (<1%) |  |
|  | gt1e | 2 (<1%) |  |
|  | gt1g^a^ | 6 (2.5%) |  |
|  | gt1l | 7 (3%) |  |
|  | gt1 unassigned | 4 (2%) |  |
| gt2 (n=5; 2%) | gt2b | 1 (<1%) | gt2d (Benin)^ref [23]^ |
|  | gt2c | 2 (<1%) | gt2g (Guinea)^ref [23]^ |
|  | gt2q | 1 (<1%) | gt2h (Guinea)^ref [23]^ |
|  | gt2 unassigned | 1 (<1%) |  |
| gt3 (n=19; 8%) | gt3a | 12 (5%) | None |
|  | gt3h | 7 (3%) |  |
| gt4 (n=110; 47%) | gt4a | 39 (17%) | gt4p (Cameroon, Uganda)^refs [22,24]^ |
|  | gt4b | 2 (<1%) | gt4q (Rwanda, Uganda)^refs [22,24]^ |
|  | gt4c | 4 (2%) | gt4s (Uganda)^ref [22]^ |
|  | gt4d | 8 (3.5%) |  |
|  | gt4f | 2 (<1%) |  |
|  | gt4g | 1 (<1%) |  |
|  | gt4k | 7 (3%) |  |
|  | gt4m | 4 (2%) |  |
|  | gt4n | 5 (2%) |  |
|  | gt4o | 4 (2%) |  |
|  | gt4r | 26 (11%) |  |
|  | gt4t | 1(<1%) |  |
|  | gt4v | 7 (3%) |  |
| gt5 (n=2; <1%) | gt5a | 2 (<1%) |  |

**^a^** One gt1g-infected patient also had a minority of sequences mapping to gt4r; this could represent co-infection with both subtypes but for analytical purposes, the patient was considered as gt1g-infected.
