# Supplementary material for: Real-World Outcomes of Direct-Acting Antiviral Treatment and Retreatment in United Kingdom–Based Patients Infected With Hepatitis C Virus Genotypes/Subtypes Endemic in Africa
Source: J Infect Dis. 2021 Mar 1;226(6):995–1004. doi: 10.1093/infdis/jiab110 (PMC9492310; doi:10.1093/infdis/jiab110)
Supplement: jiab110_suppl_Supplementary_Table_5 [file jiab110_suppl_supplementary_table_5.docx]

**Supplementary Table 5.** Statistical analysis of the factors associated with SVR.

|  | **SVR** | **non-SVR** | **p-value (univariate)** |
| --- | --- | --- | --- |
| **Average Age**  **(range)** | 55  (47-63) | 57  (51-63) | *0.47* |
|  | **% SVR  (n)** | **% non-SVR (n)** | **p-value (univariate)** |
| **Route of transmission** |  |  | *1* |
| Blood products | 88 (36) | 12 (5) |  |
| Injecting drug use | 80 (12) | 20 (3) |  |
| Sexual partner | 100  (3) | 0 (0) |  |
| Unknown | 82 (85) | 18 (18) |  |
| **Previous treatment** |  |  |  |
| Treatment naïve | 88  (85) | 12  (11) | ***0.046**** |
| Treatment experienced | 77  (51) | 23  (15) |  |
| Previous IFN-based therapy | 82 (49) | 18 (11) | *0.5* |
| No previous IFN-based therapy | 85  (87) | 15  (15) |  |
| Previous NS3-based therapy | 91 (10) | 9 (1) | *1* |
| No previous NS3-based therapy | 83  (126) | 17  (25) |  |
| Previous NS5A-based therapy | 58 (7) | 42 (5) | ***0.022**** |
| No previous NS5A-based therapy | 86  (129) | 14  (21) |  |
| Previous NS5B-based therapy | 58 (7) | 42 (5) | ***0.022**** |
| No previous NS5B-based therapy | 86  (129) | 14  (21) |  |
| **Baseline Viral Load** | 875167  (234075-2384701) | 1047145  (671403-2557500) | *0.42* |
| **Gender^a^** |  |  | *0.5* |
| Female | 87 (49) | 13 (7) |  |
| Male | 82 (86) | 18 (19) |  |
| **Ethnicity** |  |  | *0.15* |
| Black African/Caribbean | 77 (62) | 23 (19) |  |
| Arabic | 93 (26) | 7 (2) |  |
| White | 90 (19) | 10 (2) |  |
| Mixed | 83 (10) | 17 (2) |  |
| Asian | 83 (5) | 17 (1) |  |
| Other | 100 (14) | 0 (0) |  |
| **Cirrhosis** |  |  | *0.2* |
| Cirrhotic | 81 (67) | 19 (16) |  |
| Non-cirrhotic | 87 (69) | 13 (10) |  |
| **Decompensated Liver Disease** |  |  | *0.061* |
| Decompensation | 71 (25) | 29 (10) |  |
| No decompensation | 87 (111) | 13 (16) |  |
| **HCC** |  |  | ***0.006***** |
| HCC | 59 (10) | 41 (7) |  |
| No HCC | 87 (126) | 13 (19) |  |
| **HIV** |  |  | *1* |
| HIV+ | 90 (9) | 10 (1) |  |
| HIV- | 84 (127) | 16 (25) |  |
| **Viral Genotype** |  |  |  |
| gt1l/gt4r | 48 (12) | 52 (13) | ***<0.0001*** *<0.0001*** ^b^** |

^a^ gender was not recorded for 1 individual

^b^ multivariate logistic regression
