# Supplementary material for: Real-World Outcomes of Direct-Acting Antiviral Treatment and Retreatment in United Kingdom–Based Patients Infected With Hepatitis C Virus Genotypes/Subtypes Endemic in Africa
Source: J Infect Dis. 2021 Mar 1;226(6):995–1004. doi: 10.1093/infdis/jiab110 (PMC9492310; doi:10.1093/infdis/jiab110)
Supplement: jiab110_suppl_Supplementary_Table_6 [file jiab110_suppl_supplementary_table_6.docx]

| **Group (n)** | **Known genotype (n)** | **Ngt1 (%)** | **Ngt2 (%)** | **Ngt3 (%)** | **Ngt4 (%)** | **Ngt5/6 (%)** |
| --- | --- | --- | --- | --- | --- | --- |
| UK, White (8419) | 7744 | 4334  (56%) | 430  (5.5%) | 2874  (37%) | 88  (1%) | 18  (<1%) |
| UK, White, PWID (5360) | 4963 | 2647 (53%) | 248 (5%) | 2045 (41%) | 22 (0.5%) | 1 (<0.1%) |
| African (319) | 233 | 97 (42%) | 5  (2%) | 19  (8%) | 110 (47%) | 2 (<1%) |

**Supplementary Table 6.** Comparison of HCV genotypes represented in the HCV Research UK cohort.
