# Supplementary material for: Real-World Outcomes of Direct-Acting Antiviral Treatment and Retreatment in United Kingdom–Based Patients Infected With Hepatitis C Virus Genotypes/Subtypes Endemic in Africa
Source: J Infect Dis. 2021 Mar 1;226(6):995–1004. doi: 10.1093/infdis/jiab110 (PMC9492310; doi:10.1093/infdis/jiab110)
Supplement: jiab110_suppl_Supplementary_Methods [file jiab110_suppl_supplementary_methods.docx]

**SUPPLEMENTARY METHODS**

***HCV Research UK Clinical Database***

HCV Research UK recruited patients attending 57 clinical sites in England, Scotland and Wales. 11,916 individuals were enrolled between 2012 and 2017, and data was recorded as both fixed data options (e.g. for ‘Probable Source of Infection’, ‘Ethnicity’, Country of Birth’ etc) and free text (e.g. ‘Co-morbidities’). The African group were selected solely by searching for countries of birth given as within the African continent. Data on ‘Probable Source of Infection’ was entered from nine dropdown options (‘Blood/Blood Products’, ‘Born Abroad’, ‘Injecting Drug Use’, ‘Known HCV^+^ partner’, ‘Perinatal Exposure’, ‘No Known Risk Factor’, ‘Data Incomplete’, ‘No Data Entry [i.e. Blank] and ‘Other’). For those with a probable source of infection indicated by ‘Other’, a free text option was available to enter information (see Supplementary Table 1). A free text option was also available for comments on treatment. There were no records of lack of adherence and therefore it was assumed that patients adhered to their course of treatment. The database was updated on an *ad hoc* basis with a final census date of 5^th^ February 2019.

***Next Generation Sequencing (NGS) by Metagenomics and Target Enrichment***

RNA was extracted from 200-400µl serum using the Agencourt RNAdvance Blood Kit (Beckman Coulter), following manufacturer's guidelines, including DNaseI treatment at room temperature for 15 minutes. cDNA was synthesised from 11µl of extracted nucleic acid using SuperScript III (Thermo Scientific) with random hexamers, followed by NEBNext Ultra II Non-Directional RNA Second Strand Synthesis Module (New England Biolabs). The Kapa LTP Library Preparation Kit for Illumina Platforms (Kapa Biosystems) was used to prepare the library starting from the end repair step through to adapter ligation. At this stage, samples were uniquely indexed using NEBNext® Multiplex Oligos for Illumina and 16 PCR cycles were performed. Amplified libraries were quantified by Qubit dsDNA HS Kit and run on the Agilent 4200 Tapestation System using High Sensitivity D5000 Screentape and Reagents (Agilent). Samples were pooled by molar concentration, extrapolated from the mass concentration and size, and sequencing performed on Illumina’s NextSeq 500 or MiSeq platforms.

Samples processed by target enrichment were prepared as described above and resulting libraries pooled according to viral load. Libraries were enriched using the NimbleGen SeqCap EZ System (Roche) following manufacturer’s guidelines. Pooled libraries were mixed with a blocking mixture including COT (Roche), xGen Universal Blockers (IDT) and hybridization buffers (Roche). Following hybridisation at 47°C for 72 hours, libraries were incubated with streptavidin capture beads at 47°C for 45 minutes, to bind probe-associated DNA targets. Samples were further washed and eluted in water, followed by on-bead PCR using KAPA HiFi HotStart Ready Mix and Post-LM-PCR Oligos (Roche) and 14 PCR cycles were performed. Enriched library pools were quantified by Qubit dsDNA HS Kit and run on the Agilent 4200 Tapestation System, followed by sequencing as described above.
